# Microbial Diversity and Non-volatile Metabolites Profile of Low-Temperature Sausage Stored at Room Temperature

**DOI:** 10.3389/fmicb.2021.711963

**Published:** 2021-08-27

**Authors:** Hongjiao Han, Mohan Li, Yanqi Peng, Zhenghan Zhang, Xiqing Yue, Yan Zheng

**Affiliations:** College of Food Science, Shenyang Agricultural University, Shenyang, China

**Keywords:** low-temperature sausage, sausage spoilage, bacterial communities, non-volatile metabolites, omics technology

## Abstract

Sausage is a highly perishable food with unique spoilage characteristics primarily because of its specific means of production. The quality of sausage during storage is determined by its microbial and metabolite changes. This study developed a preservative-free low-temperature sausage model and coated it with natural casing. We characterized the microbiota and non-volatile metabolites in the sausage after storage at 20°C for up to 12 days. *Bacillus velezensis* was the most prevalent species observed after 4 days. Lipids and lipid-like molecules, organoheterocyclic compounds, and organic acids and their derivatives were the primary non-volatile metabolites. The key non-volatile compounds were mainly involved in protein catabolism and β-lipid oxidation. These findings provide useful information for the optimization of sausage storage conditions.

## Introduction

Low-temperature sausage is widely consumed throughout the world. The cooking process is centered on a temperature between 68 and 75°C, which retains the highest levels of quality and nutrition (Toldrá, 2017; Yang et al., 2020). However, low-temperature sausage is highly perishable because of incomplete sterilization and its natural casing (Salinas et al., 2014; Lv et al., 2021). To date, the general methods to reduce the spoilage and the maintain quality of meat products have been developed, such as the addition of preservatives (Pellissery et al., 2020), vacuum packaging, and chilled storage (Gammariello et al., 2015; Schumann and Schmid, 2018). Unfortunately, the unique appearance and quality of low-temperature sausage is detrimentally affected when stored under chilled conditions with vacuum packaging (Dong et al., 2020; Lv et al., 2021). In addition, many of these meat products are subjected to room temperature conditions and air-packaging when circulated among consumers, especially if the transportation and the cold chain are imperfect. Moreover, consumers often prefer natural, fresh, and minimally processed food with fewer additives (Pini et al., 2020). To meet these demands, our study developed a low-temperature sausage model without added preservatives that could be stored at room temperature without vacuum.

To improve the quality and safety of low-temperature sausage at room temperature, it is important to understand the changes that occur in the microbial succession within the product during storage. It has been reported that *Staphylococcus*, *Pseudomonas*, and *Acinetobacter* are the predominant bacteria contributing to the spoilage of meats at room temperature (Lv et al., 2021). Apart from these reports, there is little information on the dominant spoilage-associated bacteria in low-temperature meat products stored at room temperature. In recent years, high-throughput sequencing (HTS) approaches based on the detection of microbial DNA have been successfully applied to the identification of microorganisms and microbial communities in meat and meat products, thus providing a better understanding of the microbial quality of meat products (Polka et al., 2015; Tian et al., 2017; Chen et al., 2020; Hu et al., 2020).

Additionally, the metabolic activities of spoilage-associated microorganisms lead to the accumulation of metabolites which promote the physical and chemical deterioration of meat products (Luong et al., 2020; Bekhit et al., 2021). Thus, there is a need to simultaneously investigate both microbial communities and metabolites during storage to comprehensively the quality evolution of meat products. Recently, metabolomics has been used for the analysis and monitoring of changes in non-volatile metabolites in stored and processed meat (Castro-Puyana et al., 2017). This strategy has been successfully applied to characterize non-volatile metabolites in various types of meat, including ovine meat (Subbaraj et al., 2016), chilled chicken (Wen et al., 2020), duck liver (Zhu et al., 2020), and duck (Wang et al., 2017). Therefore, the comprehensive understanding of non-volatile metabolites in low-temperature meat products could be explored by this strategy.

In this study, we investigated the changes in bacterial succession combined with physicochemical properties and non-metabolite changes of low-temperature sausage during storage at 20°C. Specifically, we aimed to investigate (i) the physicochemical changes in the sausage based on total viable counts, total volatile basic nitrogen (TVB-N), color, and pH, (ii) microbiota changes using 16S rDNA gene analysis, and (iii) the profiles of non-volatile metabolites in low-temperature sausage by untargeted LC-MS/MS-based metabolomics.

## Materials and Methods

### Preparation of Low-Temperature Sausage

The low-temperature sausage were supplied by a local Chinese meat factory in Fuxin, Liaoning Province, China, and had been manufactured in accordance with Chinese food safety standards based on the method of Jin et al. (2017) with some modifications. A total of five independent batches were collected. The formulation included pork belly (95%, the ratio of fat to skin is about 3:1), salt (2.5%), starch (2%), sugar [0.4% (wt/wt)], and ice water (0.5–1.5%). The meats were minced in a grinder with an 8-mm plate and mixed with other supplementary material. Then, the mixture was stuffed into 30–35 mm diameter porcine natural casings, 10–12 cm long. Thereafter, the raw sausages were steamed at a temperature of 70–80°C for approximately 30 min until the internal temperature reached 74°C (Walton and McCarthy, 2007). When the steamed sausage had returned to room temperature, it was rapidly transferred to the laboratory within 2 h in a portable constant-temperature incubator (BX-10B, Kai Hang Instrument Co., Ltd., Changzhou, China). The sausages were then packed under aseptic laboratory conditions into permeable polyethylene bags (200 × 300 mm) and stored in a constant temperature humidity chamber (LHS-50CL, Shanghai Yiheng Instruments Co., Ltd., Shanghai, China) at 20°C and humidity of 50 ± 5%. The sausages used for physicochemical, metabolites, and microbiological analysis were harvested at 0, 2, 4, 6, 8, 10, and 12 days during storage.

### Physicochemical Analysis

Ten grams of sausage were ground to a puree in 90 ml deionized water according to the method of Chen et al. (2019) with some modifications. After multiple leaching and filtering in a stomacher (Pro-media, SH-2M) with a filter net, the pH of the supernatant was determined with a pH meter (PHS-3C pH Meters, Shanghai Kang Yi Instrument Co., Ltd., China).

The colors of the sausage samples were measured by a Minolta CM-2600d/2500d Chroma meter (Minolta Camera Co., Ltd., Osaka, Japan) using the standard white version for calibration before the measurement. Measurements were made perpendicular to the individual sample surface at 10 different positions. The measured areas on the sausage surface were randomly selected, sampling as much of the surface as possible and avoiding areas with obvious visual defects such as slime or exudate. The results were shown as lightness (*L*^∗^), redness (*a*^∗^), and yellowness (*b*^∗^) values.

The TVB-N content was measured according to the Chinese National Food Safety Standard method GB 5009.228-2016 [National Health and Family Planning Committee of China (NHFPC), 2016]. Briefly, a 10 g sample was homogenized in 70 mL of distilled water for 30 min and then filtered. Then, 10 mL of the filtrate was transferred to a distillation tube. After adding 5 mL of magnesium oxide suspension (10 g/L), the distillation tube was connected to an automatic Kjeldahl (K1100, Hanon Instruments Co., Ltd., Jinan, China), according to the instructions. The distillate was collected using a boric acid solution (20g/L) and then titrated with hydrochloric acid standard titration solution. Finally, the TVB-N content was calculated as:

$$T\,V\,B\,\text{-}\,N\,\left(m\,g/100\,g\right)=\frac{\left(V\,1-V\,2\right)\times \text{c}\times 14}{\text{m}}\times 10$$

Where: V1 denotes the titration volume of the hydrochloric acid consumed by the sample (mL), V2 denotes the titration volume of the blank sample (mL), c denotes the actual concentration of hydrochloric acid (mol/L), and m denotes the sample weight (g).

### Microbiological Analyses

#### Total Viable Counts

The total viable counts were determined as described by Li X. et al. (2019) with some modifications. Twenty-five grams of sausage sample were mixed with 225 mL sterile saline solution (NaCl, 8.5 g/L) and homogenized in the stomacher machine. Serial decimal dilutions were prepared, and 100 μl aliquots of the sample suspension were spread in triplicate on the Plate Count Agar (Beijing Aoboxing Bio-Tech Co., Ltd). The plates were incubated for 48 ± 2 h at 36 ± 1°C. The results were calculated and expressed as the means of log CFU/g.

#### Bacterial DNA Extraction and Sequencing

Total DNA was extracted using the CTAB method following an earlier reported method (Via and Falkinham, 1995; Brandfass and Karlovsky, 2008). The DNA purity was checked on 1% agarose gel electrophoresis, and the DNA concentration was measured using an ultraviolet spectrophotometer. The V3–V4 region of 16S ribosomal DNA was amplified used a pair of universal primers [341F(5′-CCTAYGGGRBGCASCAG-3′); 806R(5′-GGACTACNNGGGTATCTAAT-3′)] with the barcode. Sequencing libraries were generated using the Ion Plus Fragment Library Kit (Thermo Fisher Scientific, Inc., Waltham, MA, United States) following the manufacturer’s recommendations. The library was then sequenced on an Ion S5^TM^ XL platform, and 400 bp/600 bp single-end reads were generated.

#### Sequencing Analysis

Quality filtering was performed using Cutadapt software (Version 1.9.1) (Martin, 2011). The data were queried in the SILVA database (Quast et al., 2013) using the UCHIME algorithm (UCHIME Algorithm)^1^ (Edgar et al., 2011) to check and remove chimeric sequences (Haas et al., 2011). Sequences with ≥97% similarity were clustered into the operational taxonomic units (OTUs) by using UPARSE software (Version 7.0.1001) (Edgar, 2013). The SILVA database^2^ was used based on the Mothur algorithm to annotate taxonomic information for each OTU representative sequence. Beta diversity on unweighted UniFrac was performed by QIIME software (Version 1.7) (Caporaso et al., 2010).

### Analysis of Non-volatile Metabolites

#### Metabolite Extraction

Metabolites were extracted as described by Wen et al. (2020) with some modifications. Briefly, 100 mg of samples were individually ground with liquid nitrogen and homogenized in 80% methanol and 0.1% formic acid, following which the mixtures were stored in an ice bath for 5 min. The samples were then centrifuged at 15,000 rpm at 4°C for 5 min. A part of the supernatant was diluted with LC-MS-grade water to a final concentration containing 60% methanol. The samples were subsequently transferred into an Eppendorf tube with a 0.22 μm filter and were centrifuged at 15,000 × *g* at 4°C for 10 min. Finally, the filtrate was collected and stored for analysis. Equivalent metabolite extracts from each sausage sample were used for quality control (QC).

#### UHPLC-MS/MS Methods

Samples were analyzed using a Vanquish UHPLC system (Thermo Fisher Scientific) coupled with an Orbitrap Q Exactive HF-X mass spectrometer (Thermo Fisher Scientific). Metabolite extracts were separated on a Hypersil Gold column (100 × 2.1 mm, 1.9 μm, 4°C, 0.2 mL/min). The eluents of the positive polarity mode consisted of eluent A (0.1% formic acid) and eluent B (methanol). The eluents of the negative polarity mode consisted of eluent A (5 mM ammonium acetate, pH 9.0) and eluent B (methanol). The solvent gradient was initially 2% B (1.5 min) then 2% to 100% B (12 min), 100% B, (14 min), 100% to 2% B (14.1 min), and 2% B (16 min).

#### Raw MS Data Processing

The original data were converted by Compound Discoverer (Version 3.0, Thermo Fisher Scientific) to perform peak alignment, peak picking, and quantitation. The peak intensities were converted to the total spectral intensity. Then, the molecular formula was predicted according to the additive ions, molecular ion peaks, and fragment ions. Following that, the peaks were matched with the mzCloud^3^ and ChemSpider^4^ databases to obtain accurate qualitative and relative quantitative results.

### Statistical Analysis

The data analysis was performed on the General Linear Model procedure of Statistix 8.1 software package (Analytical Software, St. Paul, MN, United States). Analysis of variance (ANOVA) with Tukey’s multiple comparisons test was used at a significance level of α = 0.05. The results were expressed as means ± standard error of the mean. Principal component analysis (PCA) and partial least squares-discriminant (PLS-DA) were completed using R-3.5.3 software. The screening of differential metabolites depended on the variable importance in the projection (VIP) > 2 and *p* < 0.05.

## Results

### Analysis of Physicochemical Properties

#### pH

**FIGURE 1:**
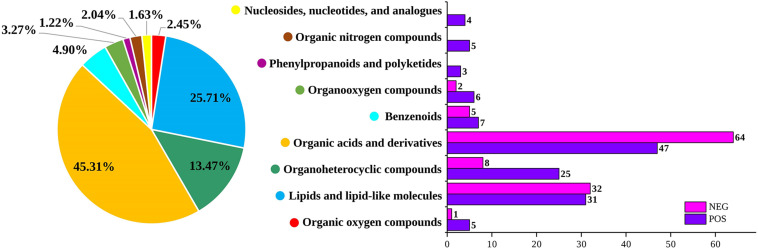
Numbers of non-volatile metabolites and their percentages in both positive and negative ion modes.

The changes in pH values during the storage of the low-temperature sausage are listed in Table 1. Before storage, the initial pH of the sausage was 7.00 ± 0.01 which finally decreased to 6.20 after being stored for 12 days. At the early storage stage (0–4 days), the pH showed a slight decrease due to lower levels of acidic substances (Lv et al., 2021). As the numbers of aerobic bacteria increased (Table 1), glucose was degraded into organic acids (Dainty et al., 1989), creating an acidic environment, leading to a sharp and significant decreasing trend between 4 and 12 days (*p* < 0.05). Additionally, it was found that organic acids and their derivatives were the predominant differential metabolites, accounting for 45.31% of the total (shown in Figure 1), confirming the decreasing pH trend. This continuous decrease in pH has been previously demonstrated and is consistent with the findings of Benson et al. (2014) and Luong et al. (2020) in pork sausage.

**TABLE 1:** Total viable counts, pH, TVB-N, and color changes in low-temperature sausage during storage.

Physicochemical analyses	Storage times (days)						
	
	0	2	4	6	8	10	12
pH	7.00 ± 0.01^a^	6.87 ± 0.01^a^	6.78 ± 0.03^b^	6.58 ± 0.02^c^	6.37 ± 0.06^d^	6.25 ± 0.04^e^	6.20 ± 0.05^f^
TVB-N (mg/100g)	6.54 ± 0.01^g^	8.67 ± 0.05^f^	13.43 ± 0.12^e^	22.37 ± 0.40^d^	25.92 ± 0.07^c^	28.00 ± 0.02^b^	35.48 ± 0.02^a^
L*	51.83 ± 0.21^a^	51.33 ± 0.17^a^	50.98 ± 0.35^b^	49.47 ± 0.29^c^	48.52 ± 0.17^d^	47.93 ± 0.33^e^	47.19 ± 0.33^f^
a*	18.21 ± 0.12^a^	17.19 ± 0.17^b^	15.15 ± 0.14^c^	14.64 ± 0.14^d^	10.72 ± 0.17^e^	10.51 ± 0.24^e^	9.32 ± 0.16^e^
b*	9.19 ± 0.07^a^	9.00 ± 0.11^a^	8.32 ± 0.10^b^	7.46 ± 0.11^c^	7.24 ± 0.12^cd^	6.83 ± 0.28^d^	5.62 ± 0.20^e^
(TVC)log CFU/g	2.25 ± 0.03^f^	3.82 ± 0.04^e^	6.82 ± 0.12^d^	8.03 ± 0.10^c^	8.70 ± 0.09^b^	8.95 ± 0.10^ab^	9.05 ± 0.10^a^

*Values are presented in the table as the mean ± standard deviation.*

*Means within the different superscript on a row indicate significant differences (*p* < 0.05).*

The results were shown as lightness (L*), redness (a*), and yellowness (b*) values.

#### Color

Color is both an effective criterion as well as an essential index to evaluate the appearance, quality, and freshness of meat products (Rubio et al., 2008). The values of *L*^∗^ and *b*^∗^ decreased continuously after 12 days’ storage but there was no significant fluctuation at the beginning of storage (0–2 days) (*p* > 0.05). The *L*^∗^-value represents the color of the sausage surface and its decrease may be responsible for moisture evaporation (Ferrini et al., 2012). The *a*^∗^-values decreased significantly (*p* < 0.05) until 6 days but did not change thereafter. The reduced *a*^∗^-values may be indicative of lipid oxidation (Yu et al., 2002). The changes in these color values indicated color deterioration in the spoiled samples.

#### TVB-N

The TVB-N value is one of the core indices to evaluate the freshness of meat products and represents the enzymatic and bacterial degradation of protein and non-protein nitrogenous substances (Chen et al., 2013). In this study, the TVB-N values increased slightly between 0 and 4 days and then increased rapidly after 6 days. Colby and Zatman (1973) reported that the accumulation of compounds contributing to the TVB-N is usually seen as a lag phase before an exponential growth period. Similarly, Tan et al. (2019) have also reported a lag phase in amine compounds in chicken meat stored at 20°C.

### Microbiological Analyses

#### Total Viable Counts

Table 1 shows the counts of total aerobic bacteria at different storage times. The initial TVC of the fresh sausage started at 2.25 CFU/g and gradually increased to a maximum of 9.05 CFU/g at the end of the storage period. The TVC increased sharply from 0 to 4 days, possibly because there are sufficient nutrients to ensure bacterial growth (Lv et al., 2021). However, the bacterial growth slowed between 6 and 12 days, which may have been due to an inhibition of growth in a low pH environment created by bacterial metabolism. Therefore, the observed constant decrease in pH was consistent with the gradual increase in bacterial growth seen between 4 and 12 days.

#### Microbial Community Structure

**FIGURE 2:**
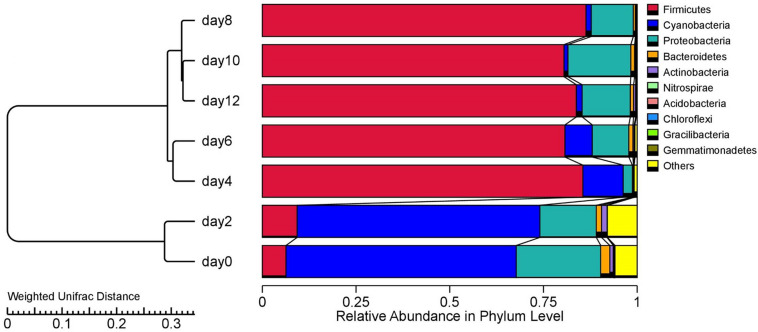
The relative abundance of bacterial phyla (top 10) at different stages of storage.

A total of 2,306, 140 high-quality bacterial sequences with an average length of 411 bp was chosen and classified into 8,487 OTUs with 97% similarity. A total of 20 phyla were identified, of which the top 10 are shown on the right side of Figure 2. To better illustrate the bacterial succession, the cluster tree analysis of the stored sausages is shown on the left side of Figure 2. The storage period was divided into three stages, with sausages stored between 0 and 2 days separated from those stored for longer periods. In the later storage period (4–12 days), there was a further separation of sausages stored between 4 and 6 days. These results suggest that the bacterial communities tended to be stable after 4 days, and few microorganisms became dominant (Li N. et al., 2019).

At the beginning of storage (0–2 days), Cyanobacteria and Proteobacteria were the most abundant phyla, accounting for 61.44% to 64.76%, and 22.52% to 15.05% of the total bacterial phyla, respectively. As reported, the initial microbial levels of meat products are strongly dependent on the processing environment (Stellato et al., 2016). Thus, the bacterial composition in the early storage period of this study may have been due to environmental issues such as meat handling and storage conditions. At 4 days, the relative abundance of Cyanobacteria and Proteobacteria decreased, while the Firmicutes increased to 85.5% of the total bacteria, and their relative abundance did not fluctuate significantly from the 6 days to the 12 days (*p* > 0.05). Similar changes in Firmicutes were observed during the storage of smoked bacon over 45 days (Li X. et al., 2019).

**FIGURE 3:**
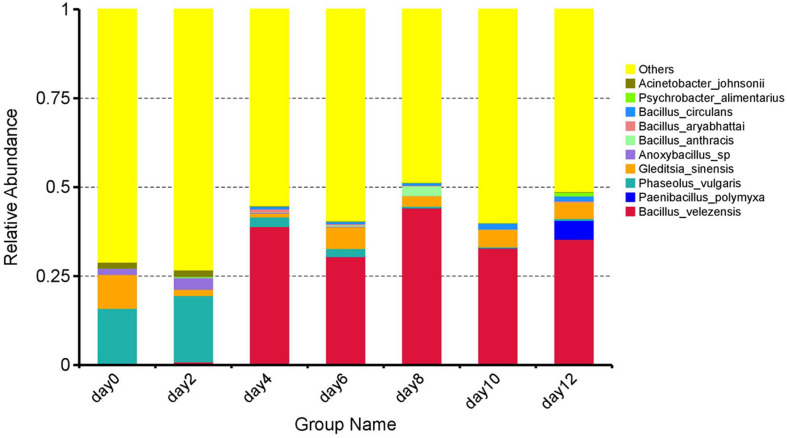
The relative abundance of bacterial species (top 10) at different stages of storage.

At the species level, an overview of the top 10 species is shown in Figure 3. It can be seen that the species composition underwent significant fluctuations. At 0 day, the predominant species were *Phaseolus vulgaris* (15.86%) and *Gleditsia sinensis* (9.52%). Although the TVC were low at the initial storage stage of 0–2 days as described above, the species proportions and abundance changed continuously. At 2 days, the dominant species was *P. vulgaris* (18.66%), while the abundance of *G. sinensis* had decreased to 1.75%. There were further changes until the composition of the microbial population gradually stabilized in the later storage stage (4–12 days). *Bacillus velezensis* was present throughout comprising more than 30% of the total population without significant fluctuation (*p* > 0.05), thus holding a relatively dominant position. As reported, *B. velezensis* was identified by Li et al. (2018) in the slaughter wastewater of a meat factory in Hunan Province, China, suggesting that the major microbial species associated with spoilage may have originated from the raw meat (Doulgeraki et al., 2012). Presumably, as a result of the mild heat treatment (68–75°C) used in this study, the microbes in the raw meat were not completely killed, allowing them to proliferate after the heat treatment. As *B. velezensis* became the predominant bacterial species from the fourth day to the end of storage suggested that *B. velezensis* was the dominant spoilage species in the sausage during the 20°C storage.

### Non-volatile Metabolites Analysis

#### Principal Component Analysis of Non-volatile Metabolites

**FIGURE 4:**
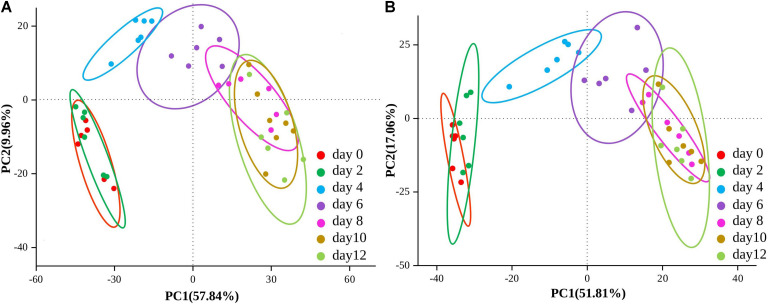
Score plots of the principal component analysis (PCA) of both positive **(A)** and negative **(B)** ion mode of non-volatile metabolites at different stages of storage.

Using untargeted LC-MS/MS-based metabolomics, a total of 2,979 peaks (1,770 from the positive mode; 1,209 from the negative mode) were identified by the mzCloud and ChemSpider databases in low-temperature sausage. Among these, 845 (480 from the positive mode; 365 from the negative mode) metabolites were annotated by the Human Metabolome Database (HMDB)^5^ (Wishart et al., 2007). To obtain a comprehensive overview of the sausage samples, PCA analysis of non-volatile metabolites was performed and is shown in Figure 4. The present PCA model could detect 67.8% (PC1 57.84% and PC2 9.96%) and 68.87% (PC1 51.81% and PC2 17.06%) variation among the samples in both the positive and negative ion modes, respectively. Moreover, it also revealed the variation and similarity of the non-volatile metabolite compositions at different storage times. Thus, the 12-day storage period was clustered into three groups: 0–2, 4–6, and 8–12 days. The microbial community as described above showed a corresponding trend of change over the storage time.

#### Profiling of Differential Non-volatile Metabolites

**FIGURE 5:**
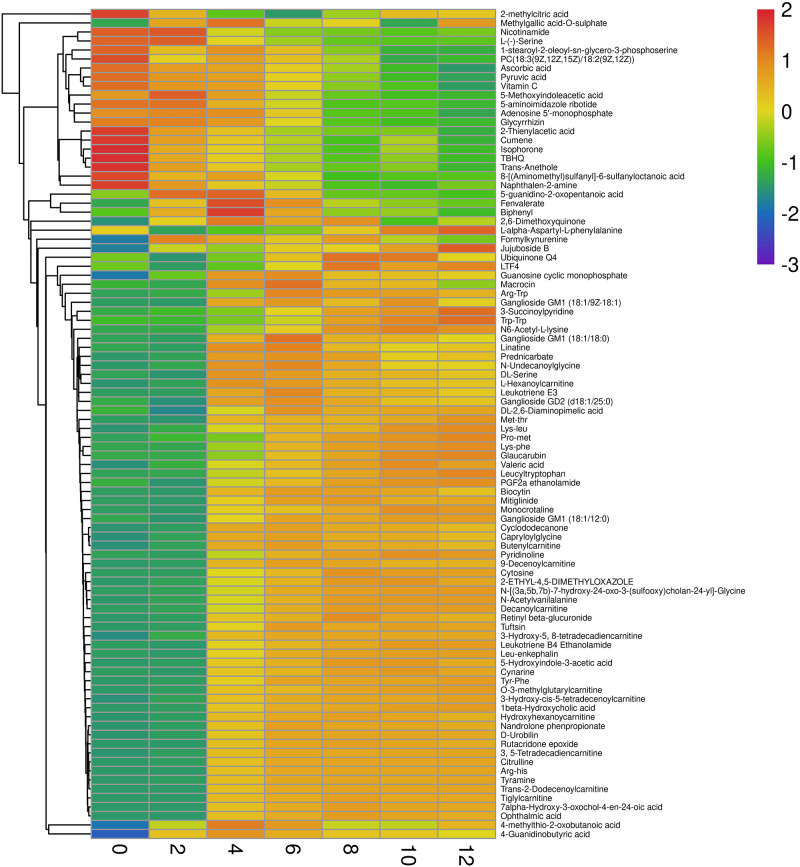
Heatmap of individual non-volatile metabolites determined in both positive and negative ion modes. The scale with the range from –3 to 2 refers to the trend of peak area after logarithmic transformation. Red and blue color indicates the high and low content of different non-volatile metabolites, respectively.

As shown in Figure 1, 245 differential non-volatile metabolites were obtained based on VIP > 2 and *p* < 0.05. These non-volatile metabolites were assigned to nine classes of 92 compounds, including organic oxygen compounds, lipids and lipid-like molecules, organoheterocyclic compounds, organic acids and their derivatives, benzenoids, organooxygen compounds, phenylpropanoids and polyketides, organic nitrogen compounds, nucleosides, nucleotides, and analogs. The comprehensive list of 245 significant metabolites at different storage times is provided in the Supplementary Tables 1–6. Organic acids and their derivatives, lipids and lipid-like molecules, and organoheterocyclic compounds were the major non-volatile metabolites identified in low-temperature sausage. Figure 5 shows a visualization of relative content changes of non-volatile metabolites in the form of a heatmap.

During storage, microorganisms and endogenous enzymes degrade proteins in the sausage to various intermediate products, such as small peptides, tripeptides, dipeptides, and single amino acids, all of which greatly affect the quality of the meat product (Spaziani et al., 2009; Berardo et al., 2017; Hanagasaki and Asato, 2018). In this study, DL-serine, L-serine, N6-acetyl-L-lysine, pyridinoline, 4-guanidinobutyric acid, and capryloylglycine were found to be the primary free amino acids in low-temperature sausage during storage and were the main contributors to the differences between the groups. 4-guanidinobutyric acid and capryloylglycine are the degradation products of arginine and the glycine precursor, respectively. The relative contents of these compounds differed across the storage stages (Figure 5). In addition, the decarboxylation of proteins or amino acids can result in the formation of biogenic amines, especially during relatively long storage periods (Lorenzo et al., 2017). As a typical biogenic amine in meat products, tyramine was identified in low-temperature sausage and was observed to increase over the storage time (Figure 5). A similar increase was previously reported in fermented sausage stored at 17 ± 2°C for 21 days (Bover-Cid et al., 2006). Another biogenic amine-related compound is formylkynurenine, formed during tryptophan degradation, was also a major contributor frequently observed in each group. Furthermore, various peptides containing Arg-His, Lys-Leu, Lys-Phe, Pro-Met, Tyr-Phe, Trp-Trp, Leu-enkephalin, and leucyltryptophan, also intermediate products of proteolysis, were identified. The peptide content was low between 0 and 2 days but increased rapidly thereafter.

In the current study, a total of nine acylcarnitines (ACs) were observed during the storage of the low-temperature sausage, namely, 3-hydroxy-cis-5-tetradecenoylcarnitine, 3-hydroxy-5, 8-tetradecadiencarnitine, O-3-methylglutarylcarnitine, hexanoylcarnitine, 3, 5-tetradecadiencarnitine, and hydroxyhexanoycarnitine. As shown in Figure 5, only hexanoylcarnitine levels were high for the first 4 days, whereafter a decreasing trend was observed. Other ACs were abundant, and their concentrations increased steadily in the later storage period of 6–12 days (Figure 5).

**FIGURE 6:**
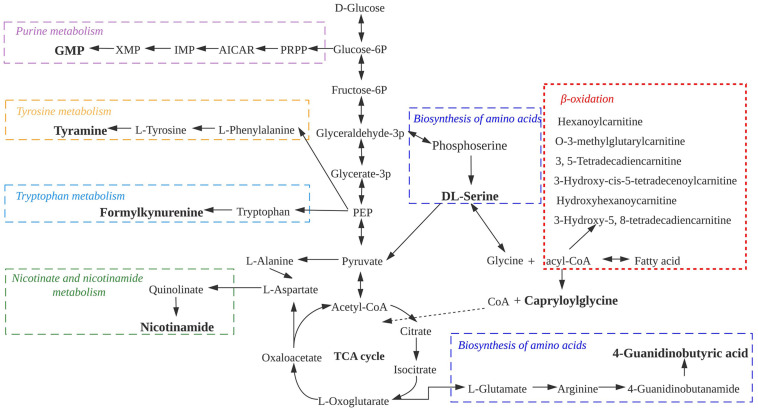
Metabolic pathways of differential non-volatile metabolites in low-temperature sausage.

To better analyze the relationship between the differential non-volatile metabolites during sausage storage, we investigated the biochemical processes responsible for their production (Figure 6). These processes included the metabolism of purine, tyrosine, and tryptophan, as well as nicotinate and nicotinamide and the biosynthesis of amino acids and β-lipid oxidation, all of which affected the changes in bacterial composition encouraging further growth and proliferation of the dominant microbial species. It appears from Figure 6 that the majority of non-volatile metabolites were associated with amino acid and bioamine metabolism. The presence of intermediate products from these pathways is usually indicative of proteolysis, and their accumulation contributes to the deterioration in meat quality (Hanagasaki and Asato, 2018; Wen et al., 2020). In addition, ACs are the principal products of β-lipid oxidation, suggesting a prominent role for β-lipid oxidation in the low-temperature sausage during storage.

## Discussion

In this study, the sausage samples were not only exposed to an open environment during production, marketing, and distribution but were also made without any preservatives. This type of perishable sausage model has rarely been reported. In the present study, *P. vulgaris* was abundant during the initial storage stage of 0–2 days, after which its abundance gradually decreased until 4 days and then dropped significantly, indicating that *P. vulgaris* was not a main contributor to sausage spoilage. This has not been previously reported. We speculate that the presence of *P. vulgaris* was understandable for the following reasons: firstly, it may have originated from contamination during the meat handling and processing, from non-sterile items such as processing surfaces and tools, or from microbiota in the meat itself (Doulgeraki et al., 2012; Ercolini et al., 2015; Hultman et al., 2015). Secondly, the excipients may have contained chloroplasts, and the plant chloroplast has a high sequence similarity to the 16S genes of bacteria (Sun et al., 2008; Hanshew et al., 2013). Its significant impact on analysis of data sets has also been acknowledged by Chelius and Triplett (2001). In addition, it is noteworthy that *Bacillus anthracis*, the etiological agent of the acute infectious disease anthrax, was identified by DNA sequencing. As shown in the Supplementary Table 7, except for 8 days, the relative abundance of *B. anthracis* over the remaining storage period remained constant with no significant change from 0.00% on 0 day. Thus, we speculate that this was a false positive based on *B. anthracis* has similarities with strains in *Bacillus* species (Rao et al., 2010). Moreover, accurate DNA-based detection of *B. anthracis* needs clean samples and is unable to detect toxins and other non-nucleic acid based samples (Rao et al., 2010; Zasada, 2020). Nevertheless, this suggests that retailers should pay attention to hygiene to ensure the safety of meat products, especially as other microorganisms such as *Bacillus circulans*, *Psychrobacter alimentarius*, and *Acinetobacter johnsonii*, are also commonly associated with food spoilage (Yucel et al., 2009; Li et al., 2014; Wang and Xie, 2020).

Notably, there was a large proportion of unassigned bacteria (“others”) in the sausage microbial community (Figure 3). This could be attributed to the fact that the sausage contained no preservatives and was also stored in the open at room temperature, leading to increased exposure to microorganisms and thus contamination. On the other hand, sausages manufactured in different settings, using different materials, processing technology, and workshops (e.g., different temperatures and humidity) will have unique, well-balanced microbial community structures and diversity. With this in mind, the microorganisms in the environment and within the food will eventually reach an equilibrium. In other words, microorganisms from both the raw materials and the environment contribute to the species composition during storage (Aquilanti et al., 2016; Liu et al., 2019). Based on the complex microbial components mentioned above, the TVC values remained constant until the end of storage, and no pathogens were detected during processing. This variability indicates the complexity and diversity of the microflora.

Regarding the non-volatile metabolites, the number of differential non-volatile metabolites increased and then decreased with prolonged storage time. This may be attributed to a similar change in bacterial succession (Lv et al., 2021). On the first 2 days, the bacterial counts were low with no apparent accumulation of non-volatile metabolites or only slight changes in their contents. After 4–6 days, when spoilage tends to become apparent, the dominant microorganisms in the meat also typically produce more metabolic by-products (Martín et al., 1998). At this stage, *B. velezensis* was predominant and increased sharply from 6 days. As has been reported, *B. velezensis* produces a number of enzymes that strongly promote organic matter decomposition (Li et al., 2018), resulting in the accumulation of organic substances such as sulfides and nitrogen (Samaei et al., 2013; Meng and Hao, 2017; Li et al., 2018). This would account for the large numbers of non-volatile metabolites observed at this stage. After this, both the bacterial succession and the accumulation of non-volatile metabolites tend to be stabilize.

In particular, ACs showed sustained accumulation in low-temperature sausage during storage. Compared with 0 day, the fold-change (FC) increased by several hundred or even thousand during storage (Supplementary Tables 1–6), implying that ACs contributed significantly to the observed to the changes in non-volatile metabolites in the sausage during storage (Wen et al., 2020). ACs are also important intermediates of lipid β-oxidation, which is typically related to the odor or flavor of sausage meat products (Toldrá et al., 2014). In addition, aerobic conditions have been shown not only to promote β-lipid oxidation (Wen et al., 2019) but also to sustain biological processes (Yu et al., 2019). Wen et al. (2019) also found that β-lipid oxidation was the most common bacterial metabolic activity in dry sausage. Therefore, we infer that β-lipid oxidation may play an essential role in the spoilage of low-temperature sausage stored at room temperature. In addition, the observed increases in amino acid, peptide, and bioamine content are indicative of proteolysis (Zhao et al., 2016). In most cases, these proteolysis markers accumulated with the storage time, especially from 6 days up to 12 days with the majority plateauing at later stages indicating that protein catabolism was maintained at a high level throughout the storage period (Frank et al., 2020). Moreover, all the peptides detected in this study are either potentially volatile compounds or the precursors of such compounds, and all had a low molecular mass of <5,000 Da (Berardo et al., 2017). Therefore, the accumulation of these peptides not only reduced the meat quality but also produced undesirable odors.

Furthermore, it was difficult to establish a direct correlation between the microbial populations and metabolites because the non-volatile metabolites are derived from both the microorganisms and the sausage itself. Overall, this paper aimed to study the chemical spoilage of low-temperature sausage from the macroscopic perspective. To our knowledge, there have been no reports regarding the changes of non-volatile metabolites in sausage over a 12 days’ storage period.

## Conclusion

The dynamic changes in the bacterial communities and non-volatile metabolites of low-temperature sausage stored at 20°C for 12 days have been reported. The changes in the physical and chemical properties of the sausage verified the spoilage process, shown by the decreases in pH, *L*^∗^, *a*^∗^, and *b*^∗^ values, and the increases in TVB-N and TVC. Firmicutes dominated the bacterial communities in the later storage period. *B. velezensis* was the dominant core bacterium that acts as a spoilage indicator and is probably associated with compounds acting as spoilage indicators. Meanwhile, different non-volatile metabolites of lipids and lipid-like molecules, organoheterocyclic compounds, and organic acids and their derivatives were present in high concentrations at different storage stages. The key non-volatile molecules were mainly involved in protein catabolism and β-lipid oxidation. This work provides valuable information for the industrialization and standardization of low-temperature meat products. However, the volatile compounds which also affect the quality of sausage during storage have not been studied. Comprehensive metabolomic analysis under different storage conditions should be carried out in the future so that these results can improve the processing of low-temperature meat products.

## Data Availability Statement

The datasets presented in this study can be found in online repositories. The names of the repository/repositories and accession number(s) can be found below: NCBI BioProject PRJNA731615; MetaboLights MTBLS2871.

## Author Contributions

HH carried out the original draft, data curation, and data analyses. HH, ZZ, and YP performed the experiments. ML and YZ provided critical comments. HH, YZ, and XY conceived and designed the idea of the study. All authors reviewed and approved the final manuscript.

## Conflict of Interest

The authors declare that the research was conducted in the absence of any commercial or financial relationships that could be construed as a potential conflict of interest.

## Publisher’s Note

All claims expressed in this article are solely those of the authors and do not necessarily represent those of their affiliated organizations, or those of the publisher, the editors and the reviewers. Any product that may be evaluated in this article, or claim that may be made by its manufacturer, is not guaranteed or endorsed by the publisher.

## Footnotes

1
http://www.drive5.com/usearch/manual/uchime_algo.html

2
https://www.arb-silva.de/

3
https://www.mzcloud.org/

4
http://www.chemspider.com/

5
http://www.hmdb.ca/
