# Supplementary material for: Microbial Diversity and Non-volatile Metabolites Profile of Low-Temperature Sausage Stored at Room Temperature
Source: Front Microbiol. 2021 Aug 27;12:711963. doi: 10.3389/fmicb.2021.711963 (PMC8430334; doi:10.3389/fmicb.2021.711963)
Supplement: Supplementary file 1 [file Data_Sheet_1.docx]

***Supplementary Material***

**Table S1.** Significant different metabolites of low-temperature sausage in 0 days and 2 days

| Metabolites | Classification | VIP | FC | P value |
| --- | --- | --- | --- | --- |
| **Formylkynurenine** | **Organic oxygen compounds** | **13.30** | **6.97** | **0.0148** |
| **4-Guanidinobutyric acid** | **Organic acids and derivatives** | **6.79** | **5.47** | **<0.01** |
| L-alpha-Aspartyl-L-phenylalanine | Organic acids and derivatives | 6.19 | 0.21 | <0.01 |
| Methylgallic acid-O-sulphate | Benzenoids | 6.08 | 3.93 | 0.0332 |
| **Guanosine cyclic monophosphate** | **Nucleosides, nucleotides, and analogues** | **5.49** | **4.00** | **<0.01** |
| 8-[(Aminomethyl)sulfanyl]-6-sulfanyloctanoic acid | Lipids and lipid-like molecules | 4.47 | 0.28 | 0.0396 |
| **3-Hydroxy-5, 8-tetradecadiencarnitine** | **Organic acids and derivatives** | **3.85** | **2.56** | **0.0262** |
| PC(18:3(9Z,12Z,15Z)/18:2(9Z,12Z)) | Lipids and lipid-like molecules | 3.60 | 0.43 | <0.01 |
| Isophorone | Organooxygen compounds | 3.50 | 0.41 | <0.01 |
| 5-guanidino-2-oxopentanoic acid | Organic acids and derivatives | 3.30 | 2.35 | <0.01 |
| 5-Methoxyindoleacetic acid | Organoheterocyclic compounds | 3.13 | 2.26 | <0.01 |
| LTF4 | Lipids and lipid-like molecules | 2.96 | 0.46 | 0.0277 |
| Valeric acid | Lipids and lipid-like molecules | 2.87 | 1.79 | <0.01 |
| **3-Hydroxy-cis-5-tetradecenoylcarnitine** | **Organic acids and derivatives** | **2.84** | **1.99** | **0.0297** |
| Cumene | Benzenoids | 2.79 | 0.53 | 0.0357 |
| fenvalerate | Lipids and lipid-like molecules | 2.68 | 1.80 | <0.01 |
| 1-stearoyl-2-oleoyl-sn-glycero-3-phosphoserine | Lipids and lipid-like molecules | 2.67 | 0.52 | <0.01 |
| Jujuboside B | Lipids and lipid-like molecules | 2.66 | 1.78 | <0.01 |
| TBHQ | Benzenoids | 2.65 | 0.49 | <0.01 |
| Naphthalen-2-amine | Benzenoids | 2.52 | 0.52 | 0.0273 |
| 4-methylthio-2-oxobutanoic acid | Lipids and lipid-like molecules | 2.42 | 1.76 | <0.01 |
| trans-Anethole | Benzenoids | 2.32 | 0.55 | <0.01 |
| Pyruvic acid | Organic acids and derivatives | 2.29 | 0.58 | 0.0104 |
| DL-2,6-Diaminopimelic acid | Organic acids and derivatives | 2.27 | 0.60 | 0.0121 |
| Vitamin C | Organoheterocyclic compounds | 2.27 | 0.59 | <0.01 |
| Ubiquinone Q4 | Lipids and lipid-like molecules | 2.22 | 0.63 | 0.0119 |
| **Capryloylglycine** | **Organic acids and derivatives** | **2.16** | **1.59** | **<0.01** |
| Ascorbic acid | Organoheterocyclic compounds | 2.14 | 0.64 | <0.01 |
| Biphenyl | Benzenoids | 2.10 | 1.66 | 0.0201 |
| 2,6-Dimethoxyquinone | Organic oxygen compounds | 2.09 | 1.54 | 0.0146 |
| 2-methylcitric acid | Organic acids and derivatives | 2.08 | 0.61 | <0.01 |
| 2-Thienylacetic acid | Organoheterocyclic compounds | 2.06 | 0.57 | 0.0327 |

**Table S2.** Significant different metabolites of low-temperature sausage in 0 days and 4 days

| Metabolites | Classification | VIP | FC | P value |
| --- | --- | --- | --- | --- |
| L-Hexanoylcarnitine | Lipids and lipid-like molecules | 4.94 | 59.33 | <0.01 |
| **Leu-enkephalin** | **Organic acids and derivatives** | **4.45** | **36.33** | **<0.01** |
| **3-Hydroxy-cis-5-tetradecenoylcarnitine** | **Organic acids and derivatives** | **4.22** | **408.41** | **<0.01** |
| **Guanosine cyclic monophosphate** | **Nucleosides, nucleotides, and analogues** | **4.21** | **28.06** | **<0.01** |
| **Nicotinamide** | **Organoheterocyclic compounds** | **4.10** | **0.04** | **<0.01** |
| **3-Hydroxy-5, 8-tetradecadiencarnitine** | **Organic acids and derivatives** | **3.96** | **279.33** | **<0.01** |
| **Formylkynurenine** | **Organic oxygen compounds** | **3.90** | **5.22** | **0.0202** |
| O-3-methylglutarylcarnitine | Organooxygen compounds | 3.71 | 164.92 | <0.01 |
| Monocrotaline | Organoheterocyclic compounds | 3.68 | 20.46 | <0.01 |
| Retinyl beta-glucuronide | Lipids and lipid-like molecules | 3.58 | 267.43 | <0.01 |
| Cynarine | Organooxygen compounds | 3.57 | 22.05 | <0.01 |
| **DL-Serine** | **Organic acids and derivatives** | **3.53** | **17.65** | <0.01 |
| **Capryloylglycine** | **Organic acids and derivatives** | **3.52** | **151.05** | <0.01 |
| **Tyramine** | **Benzenoids** | **3.51** | **182.01** | <0.01 |
| Ganglioside GD2 (d18:1/25:0) | Lipids and lipid-like molecules | 3.37 | 13.53 | <0.01 |
| trans-2-Dodecenoylcarnitine | Organic acids and derivatives | 3.23 | 126.46 | <0.01 |
| Leukotriene B4 Ethanolamide | Organic nitrogen compounds | 3.20 | 16.40 | <0.01 |
| N-Undecanoylglycine | Organic acids and derivatives | 3.07 | 13.58 | <0.01 |
| Ganglioside GM1 (18:1/12:0) | Lipids and lipid-like molecules | 3.06 | 18.12 | <0.01 |
| 3, 5-Tetradecadiencarnitine | Lipids and lipid-like molecules | 3.06 | 106.02 | <0.01 |
| Prednicarbate | Lipids and lipid-like molecules | 2.97 | 84.06 | <0.01 |
| **arg-his** | Organic acids and derivatives | 2.86 | 60.51 | <0.01 |
| Hydroxyhexanoycarnitine | Organic acids and derivatives | 2.85 | 66.80 | <0.01 |
| Leukotriene E3 | Lipids and lipid-like molecules | 2.77 | 9.75 | <0.01 |
| **Cytosine** | **Organoheterocyclic compounds** | **2.75** | **10.41** | **<0.01** |
| 5-Hydroxyindole-3-acetic acid | Organoheterocyclic compounds | 2.74 | 11.53 | <0.01 |
| Tiglylcarnitine | Lipids and lipid-like molecules | 2.72 | 9.67 | <0.01 |
| D-Urobilin | Organoheterocyclic compounds | 2.69 | 47.01 | <0.01 |
| **4-Guanidinobutyric acid** | **Organic acids and derivatives** | **2.67** | **8.02** | **<0.01** |
| ophthalmic acid | Organic acids and derivatives | 2.64 | 8.78 | <0.01 |
| Methylgallic acid-O-sulphate | Benzenoids | 2.60 | 5.65 | <0.01 |
| 9-Decenoylcarnitine | Organic acids and derivatives | 2.58 | 46.31 | <0.01 |
| Rutacridone epoxide | Organoheterocyclic compounds | 2.56 | 8.64 | <0.01 |
| Cyclododecanone | Organic acids and derivatives | 2.55 | 41.28 | <0.01 |
| Ganglioside GM1 (18:1/9Z-18:1) | Lipids and lipid-like molecules | 2.55 | 7.59 | <0.01 |
| Tuftsin | Organic acids and derivatives | 2.54 | 34.76 | <0.01 |
| **Tyr-Phe** | Organic acids and derivatives | 2.51 | 38.37 | <0.01 |
| Butenylcarnitine | Organic acids and derivatives | 2.48 | 38.24 | <0.01 |
| Mitiglinide | Phenylpropanoids and polyketides | 2.46 | 8.00 | <0.01 |
| 7alpha-Hydroxy-3-oxochol-4-en-24-oic acid | Lipids and lipid-like molecules | 2.40 | 33.56 | <0.01 |
| **lys-leu** | Organic acids and derivatives | 2.37 | 24.59 | <0.01 |
| **L-(-)-Serine** | **Organic acids and derivatives** | **2.37** | **0.15** | **<0.01** |

**Table S2. (continued)**

| Metabolites | Classification | VIP | FC | P value |
| --- | --- | --- | --- | --- |
| Ganglioside GM1 (18:1/18:0) | Lipids and lipid-like molecules | 2.34 | 6.57 | <0.01 |
| linatine | Organic acids and derivatives | 2.31 | 6.79 | <0.01 |
| Biocytin | Organic acids and derivatives | 2.28 | 6.97 | <0.01 |
| 1beta-Hydroxycholic acid | Lipids and lipid-like molecules | 2.21 | 30.07 | <0.01 |
| Nandrolone phenpropionate | Lipids and lipid-like molecules | 2.17 | 23.95 | <0.01 |
| met-thr | Organic acids and derivatives | 2.12 | 5.41 | <0.01 |
| Citrulline | Organic acids and derivatives | 2.10 | 23.33 | <0.01 |
| N-[(3a,5b,7b)-7-hydroxy-24-oxo-3-(sulfooxy)cholan-24-yl]-Glycine | Lipids and lipid-like molecules | 2.09 | 23.38 | <0.01 |
| Macrocin | Lipids and lipid-like molecules | 2.04 | 5.00 | <0.01 |

**Table S3.** Significant different metabolites of low-temperature sausage in 0 days and 6 days

| Metabolites | Classification | VIP | FC | P value |
| --- | --- | --- | --- | --- |
| **Leu-enkephalin** | **Organic acids and derivatives** | **4.10** | **117.09** | **<0.01** |
| **3-Hydroxy-cis-5-tetradecenoylcarnitine** | **Organic acids and derivatives** | **3.66** | **958.15** | **<0.01** |
| **Nicotinamide** | **Organoheterocyclic compounds** | **3.65** | **0.01** | **<0.01** |
| L-Hexanoylcarnitine | Lipids and lipid-like molecules | 3.36 | 57.20 | <0.01 |
| **3-Hydroxy-5, 8-tetradecadiencarnitine** | **Organic acids and derivatives** | **3.36** | **517.15** | **<0.01** |
| Ganglioside GM1 (18:1/12:0) | Lipids and lipid-like molecules | 3.35 | 62.17 | <0.01 |
| Retinyl beta-glucuronide | Lipids and lipid-like molecules | 3.27 | 620.76 | <0.01 |
| **Pyridinoline** | **Organic acids and derivatives** | **3.22** | **69.66** | **<0.01** |
| **Tyramine** | **Benzenoids** | **3.11** | **391.99** | **<0.01** |
| **Guanosine cyclic monophosphate** | **Nucleosides, nucleotides, and analogues** | **3.09** | **34.32** | **<0.01** |
| Leukotriene B4 Ethanolamide | Organic nitrogen compounds | 2.95 | 31.80 | <0.01 |
| trans-2-Dodecenoylcarnitine | Organic acids and derivatives | 2.88 | 231.82 | <0.01 |
| Cynarine | Organooxygen compounds | 2.85 | 32.28 | <0.01 |
| O-3-methylglutarylcarnitine | Lipids and lipid-like molecules | 2.78 | 225.55 | <0.01 |
| **Capryloylglycine** | **Organic acids and derivatives** | **2.77** | **196.87** | **<0.01** |
| 3, 5-Tetradecadiencarnitine | Organic acids and derivatives | 2.76 | 187.84 | <0.01 |
| Monocrotaline | Organoheterocyclic compounds | 2.65 | 27.44 | <0.01 |
| Tuftsin | Organic acids and derivatives | 2.62 | 144.74 | <0.01 |
| **arg-his** | Organic acids and derivatives | 2.57 | 122.18 | <0.01 |
| D-Urobilin | Organoheterocyclic compounds | 2.55 | 115.99 | <0.01 |
| **Cytosine** | **Organoheterocyclic compounds** | **2.54** | **20.11** | **<0.01** |
| **Formylkynurenine** | **Organic oxygen compounds** | **2.53** | **4.13** | **0.0264** |
| Ganglioside GD2 (d18:1/25:0) | Lipids and lipid-like molecules | 2.51 | 17.44 | <0.01 |
| **Tyr-Phe** | Organic acids and derivatives | 2.48 | 109.19 | <0.01 |
| 5-Hydroxyindole-3-acetic acid | Organoheterocyclic compounds | 2.45 | 17.27 | <0.01 |
| N-[(3a,5b,7b)-7-hydroxy-24-oxo-3-(sulfooxy)cholan-24-yl]-Glycine | Lipids and lipid-like molecules | 2.41 | 105.05 | <0.01 |
| Mitiglinide | Phenylpropanoids and polyketides | 2.38 | 16.74 | <0.01 |
| Hydroxyhexanoycarnitine | Organic acids and derivatives | 2.38 | 98.54 | <0.01 |
| Leukotriene E3 | Lipids and lipid-like molecules | 2.35 | 15.15 | <0.01 |
| **DL-Serine** | **Organic acids and derivatives** | **2.34** | **15.87** | **<0.01** |
| N-Undecanoylglycine | Organic acids and derivatives | 2.31 | 15.31 | <0.01 |
| **lys-leu** | Organic acids and derivatives | 2.31 | 79.94 | <0.01 |
| 9-Decenoylcarnitine | Organic acids and derivatives | 2.30 | 77.26 | <0.01 |
| Rutacridone epoxide | Organoheterocyclic compounds | 2.25 | 13.18 | <0.01 |
| **pro-met** | Organic acids and derivatives | 2.23 | 14.87 | <0.01 |
| Ganglioside GM1 (18:1/18:0) | Lipids and lipid-like molecules | 2.22 | 13.48 | <0.01 |
| Prednicarbate | Lipids and lipid-like molecules | 2.22 | 101.18 | <0.01 |
| 7alpha-Hydroxy-3-oxochol-4-en-24-oic acid | Lipids and lipid-like molecules | 2.19 | 58.56 | <0.01 |
| **L-(-)-Serine** | **Organic acids and derivatives** | **2.18** | **0.08** | **<0.01** |
| **Lys-phe** | Organic acids and derivatives | 2.17 | 13.39 | <0.01 |
| Glaucarubin | Lipids and lipid-like molecules | 2.14 | 14.56 | <0.01 |
| Tiglylcarnitine | Lipids and lipid-like molecules | 2.14 | 12.07 | <0.01 |

**Table S3. (continued)**

| Metabolites | Classification | VIP | FC | P value |
| --- | --- | --- | --- | --- |
| ophthalmic acid | Organic acids and derivatives | 2.08 | 11.32 | <0.01 |
| PGF2a ethanolamide | Lipids and lipid-like molecules | 2.07 | 11.09 | <0.01 |
| 1beta-Hydroxycholic acid | Lipids and lipid-like molecules | 2.06 | 55.40 | <0.01 |
| N-Acetylvanilalanine | Organic acids and derivatives | 2.06 | 52.27 | <0.01 |
| Butenylcarnitine | Organic acids and derivatives | 2.06 | 50.57 | <0.01 |
| Cyclododecanone | Organooxygen compounds | 2.06 | 48.68 | <0.01 |
| Arg-Trp | Organic acids and derivatives | 2.05 | 12.01 | <0.01 |
| Leucyltryptophan | Organic acids and derivatives | 2.03 | 46.50 | <0.01 |
| decanoylcarnitine | Lipids and lipid-like molecules | 2.00 | 54.46 | <0.01 |

**Table S4.** Significant different metabolites of low-temperature sausage in 0 days and 8 days

| Metabolites | Classification | VIP | FC | P value |
| --- | --- | --- | --- | --- |
| **Leu-enkephalin** | **Organic acids and derivatives** | **3.56** | **163.77** | **<0.01** |
| Retinyl beta-glucuronide | Lipids and lipid-like molecules | 3.47 | 2201.00 | <0.01 |
| **3-Hydroxy-cis-5-tetradecenoylcarnitine** | **Organic acids and derivatives** | **3.34** | **1225.37** | **<0.01** |
| **Pyridinoline** | **Organic acids and derivatives** | **3.30** | **112.47** | **<0.01** |
| **Nicotinamide** | **Organoheterocyclic compounds** | **3.15** | **0.01** | **<0.01** |
| **3-Hydroxy-5, 8-tetradecadiencarnitine** | **Organic acids and derivatives** | **2.99** | **583.09** | **<0.01** |
| O-3-methylglutarylcarnitine | Lipids and lipid-like molecules | 2.85 | 445.07 | <0.01 |
| **Tyramine** | **Benzenoids** | **2.84** | **458.06** | **<0.01** |
| Ganglioside GM1 (18:1/12:0) | Lipids and lipid-like molecules | 2.75 | 52.60 | <0.01 |
| Monocrotaline | Organoheterocyclic compounds | 2.73 | 51.54 | <0.01 |
| Leukotriene B4 Ethanolamide | Organic nitrogen compounds | 2.64 | 46.32 | <0.01 |
| Cynarine | Organooxygen compounds | 2.61 | 50.72 | <0.01 |
| trans-2-Dodecenoylcarnitine | Organic acids and derivatives | 2.57 | 253.77 | <0.01 |
| **Cytosine** | **Organoheterocyclic compounds** | **2.51** | **40.44** | **<0.01** |
| L-Hexanoylcarnitine | Lipids and lipid-like molecules | 2.49 | 36.23 | <0.01 |
| 3, 5-Tetradecadiencarnitine | Organic acids and derivatives | 2.44 | 192.13 | <0.01 |
| **arg-his** | Organic acids and derivatives | 2.34 | 138.82 | <0.01 |
| N-[(3a,5b,7b)-7-hydroxy-24-oxo-3-(sulfooxy)cholan-24-yl]-Glycine | Lipids and lipid-like molecules | 2.33 | 148.33 | <0.01 |
| **Capryloylglycine** | **Organic acids and derivatives** | **2.31** | **138.37** | **<0.01** |
| **5-Hydroxyindole-3-acetic acid** | **Organoheterocyclic compounds** | **2.31** | **26.62** | **<0.01** |
| Hydroxyhexanoycarnitine | Organic acids and derivatives | 2.25 | 124.57 | <0.01 |
| **Tyr-Phe** | Organic acids and derivatives | 2.23 | 114.44 | <0.01 |
| N6-Acetyl-L-lysine | Organic acids and derivatives | 2.22 | 25.56 | <0.01 |
| **pro-met** | Organic acids and derivatives | 2.21 | 23.03 | <0.01 |
| D-Urobilin | Organoheterocyclic compounds | 2.21 | 109.73 | <0.01 |
| Glaucarubin | Lipids and lipid-like molecules | 2.21 | 23.21 | <0.01 |
| **Formylkynurenine** | **Organic oxygen compounds** | **2.17** | **5.66** | **0.0198** |
| Tuftsin | Organic acids and derivatives | 2.16 | 104.27 | <0.01 |
| Leucyltryptophan | Organic acids and derivatives | 2.15 | 99.29 | <0.01 |
| **lys-leu** | Organic acids and derivatives | 2.14 | 90.39 | <0.01 |
| **L-(-)-Serine** | **Organic acids and derivatives** | **2.14** | **0.05** | **<0.01** |
| **Lys-phe** | Organic acids and derivatives | 2.09 | 19.59 | <0.01 |
| 7alpha-Hydroxy-3-oxochol-4-en-24-oic acid | Lipids and lipid-like molecules | 2.09 | 85.40 | <0.01 |
| N-Acetylvanilalanine | Organic acids and derivatives | 2.04 | 83.03 | <0.01 |
| decanoylcarnitine | Lipids and lipid-like molecules | 2.04 | 84.90 | <0.01 |
| 2-ETHYL-4,5-DIMETHYLOXAZOLE | Organoheterocyclic compounds | 2.04 | 88.95 | <0.01 |
| Mitiglinide | Phenylpropanoids and polyketides | 2.03 | 18.35 | <0.01 |

**Table S5.** Significant different metabolites of low-temperature sausage in 0 days and 10 days

| Metabolites | Classification | VIP | FC | P value |
| --- | --- | --- | --- | --- |
| **Pyridinoline** | **Organic acids and derivatives** | **3.47** | **184.93** | **<0.01** |
| **Leu-enkephalin** | **Organic acids and derivatives** | **3.42** | **168.22** | **<0.01** |
| **Nicotinamide** | **Organoheterocyclic compounds** | **3.22** | **0.01** | **<0.01** |
| **3-Hydroxy-cis-5-tetradecenoylcarnitine** | **Organic acids and derivatives** | **3.15** | **1039.99** | **<0.01** |
| Retinyl beta-glucuronide | Lipids and lipid-like molecules | 3.10 | 915.24 | <0.01 |
| Monocrotaline | Organoheterocyclic compounds | 2.97 | 89.60 | <0.01 |
| O-3-methylglutarylcarnitine | Lipids and lipid-like molecules | 2.88 | 536.37 | <0.01 |
| **3-Hydroxy-5, 8-tetradecadiencarnitine** | **Organic acids and derivatives** | **2.83** | **491.61** | **<0.01** |
| Ganglioside GM1 (18:1/12:0) | Lipids and lipid-like molecules | 2.79 | 73.43 | <0.01 |
| **Tyramine** | **Benzenoids** | **2.74** | **406.78** | **<0.01** |
| Cynarine | Organooxygen compounds | 2.71 | 61.63 | <0.01 |
| Leukotriene B4 Ethanolamide | Organic nitrogen compounds | 2.59 | 58.92 | <0.01 |
| trans-2-Dodecenoylcarnitine | Organic acids and derivatives | 2.48 | 255.64 | <0.01 |
| **lys-leu** | Organic acids and derivatives | 2.42 | 186.79 | <0.01 |
| Glaucarubin | Lipids and lipid-like molecules | 2.41 | 38.25 | <0.01 |
| 3, 5-Tetradecadiencarnitine | Organic acids and derivatives | 2.41 | 218.47 | <0.01 |
| **Cytosine** | **Organoheterocyclic compounds** | **2.39** | **36.16** | **<0.01** |
| N-[(3a,5b,7b)-7-hydroxy-24-oxo-3-(sulfooxy)cholan-24-yl]-Glycine | Lipids and lipid-like molecules | 2.36 | 175.42 | <0.01 |
| Leucyltryptophan | Organic acids and derivatives | 2.35 | 167.35 | <0.01 |
| N6-Acetyl-L-lysine | Organic acids and derivatives | 2.30 | 32.58 | <0.01 |
| **arg-his** | Organic acids and derivatives | 2.27 | 136.21 | <0.01 |
| **Capryloylglycine** | **Organic acids and derivatives** | **2.22** | **128.55** | **<0.01** |
| **Trp-Trp** | Organic acids and derivatives | 2.21 | 28.30 | <0.01 |
| **pro-met** | Organic acids and derivatives | 2.21 | 28.67 | <0.01 |
| 5-Hydroxyindole-3-acetic acid | Organoheterocyclic compounds | 2.21 | 27.46 | <0.01 |
| Tuftsin | Organic acids and derivatives | 2.20 | 124.97 | <0.01 |
| D-Urobilin | Organoheterocyclic compounds | 2.16 | 113.04 | <0.01 |
| Hydroxyhexanoycarnitine | Organic acids and derivatives | 2.16 | 113.53 | <0.01 |
| **Tyr-Phe** | Organic acids and derivatives | 2.14 | 109.19 | <0.01 |
| **Lys-phe** | Organic acids and derivatives | 2.12 | 24.17 | <0.01 |
| **L-(-)-Serine** | **Organic acids and derivatives** | **2.12** | **0.04** | **<0.01** |
| L-Hexanoylcarnitine | Lipids and lipid-like molecules | 2.10 | 23.54 | <0.01 |
| 2-ETHYL-4,5-DIMETHYLOXAZOLE | Organoheterocyclic compounds | 2.05 | 89.45 | <0.01 |
| N-Acetylvanilalanine | Organic acids and derivatives | 2.05 | 87.19 | <0.01 |
| Pyruvic acid | Organic acids and derivatives | 2.04 | 0.05 | <0.01 |
| Valeric acid | Lipids and lipid-like molecules | 2.02 | 84.28 | <0.01 |
| PGF2a ethanolamide | Lipids and lipid-like molecules | 2.00 | 21.63 | <0.01 |
| decanoylcarnitine | Lipids and lipid-like molecules | 2.00 | 86.26 | <0.01 |

**Table S6.** Significant different metabolites of low-temperature sausage in 0 days and 12 days

| Metabolites | Classification | VIP | FC | P value |
| --- | --- | --- | --- | --- |
| **3-Hydroxy-cis-5-tetradecenoylcarnitine** | **Organic acids and derivatives** | **3.30** | **1514.49** | **<0.01** |
| **Leu-enkephalin** | **Organic acids and derivatives** | **3.11** | **165.06** | **<0.01** |
| Retinyl beta-glucuronide | Lipids and lipid-like molecules | 2.88 | 592.55 | <0.01 |
| **Pyridinoline** | **Organic acids and derivatives** | **2.87** | **157.17** | **<0.01** |
| **3-Hydroxy-5, 8-tetradecadiencarnitine** | **Organic acids and derivatives** | **2.84** | **506.48** | **<0.01** |
| **Nicotinamide** | **Organoheterocyclic compounds** | **2.83** | **0.01** | **<0.01** |
| O-3-methylglutarylcarnitine | Lipids and lipid-like molecules | 2.82 | 547.35 | <0.01 |
| Monocrotaline | Organoheterocyclic compounds | 2.78 | 78.19 | <0.01 |
| **Tyramine** | **Benzenoids** | **2.70** | **412.54** | **<0.01** |
| Leukotriene B4 Ethanolamide | Organic nitrogen compounds | 2.56 | 56.67 | <0.01 |
| Ganglioside GM1 (18:1/12:0) | Lipids and lipid-like molecules | 2.56 | 73.02 | <0.01 |
| trans-2-Dodecenoylcarnitine | Organic acids and derivatives | 2.52 | 266.48 | <0.01 |
| Leucyltryptophan | Organic acids and derivatives | 2.45 | 222.25 | <0.01 |
| **lys-leu** | Organic acids and derivatives | 2.45 | 224.38 | <0.01 |
| 3, 5-Tetradecadiencarnitine | Organic acids and derivatives | 2.44 | 221.17 | <0.01 |
| Cynarine | Organooxygen compounds | 2.44 | 44.93 | <0.01 |
| N-[(3a,5b,7b)-7-hydroxy-24-oxo-3-(sulfooxy)cholan-24-yl]-Glycine | Lipids and lipid-like molecules | 2.40 | 203.40 | <0.01 |
| Glaucarubin | Lipids and lipid-like molecules | 2.38 | 42.75 | <0.01 |
| **pro-met** | Organic acids and derivatives | 2.31 | 36.90 | <0.01 |
| Pyruvic acid | Organic acids and derivatives | 2.28 | 0.03 | <0.01 |
| **Trp-Trp** | Organic acids and derivatives | 2.27 | 42.82 | <0.01 |
| Vitamin C | Organoheterocyclic compounds | 2.26 | 0.03 | <0.01 |
| **Tyr-Phe** | Organic acids and derivatives | 2.25 | 142.28 | <0.01 |
| **arg-his** | Organic acids and derivatives | 2.23 | 130.46 | <0.01 |
| **Cytosine** | **Organoheterocyclic compounds** | **2.21** | **32.28** | **<0.01** |
| 3-Succinoylpyridine | Organic acids and derivatives | 2.18 | 29.03 | <0.01 |
| **Lys-phe** | Organic acids and derivatives | 2.15 | 28.70 | <0.01 |
| Tuftsin | Organic acids and derivatives | 2.14 | 116.40 | <0.01 |
| 5-aminoimidazole ribotide | Organic oxygen compounds | 2.13 | 0.06 | <0.01 |
| **Capryloylglycine** | **Organic acids and derivatives** | **2.06** | **96.94** | **<0.01** |
| D-Urobilin | Organoheterocyclic compounds | 2.06 | 98.96 | <0.01 |
| **N6-Acetyl-L-lysine** | **Organic acids and derivatives** | **2.05** | **24.33** | **<0.01** |
| Adenosine 5'-monophosphate | Nucleosides, nucleotides, and analogues | 2.04 | 0.05 | <0.01 |
| Glycyrrhizin | Lipids and lipid-like molecules | 2.03 | 0.05 | <0.01 |
| **L-(-)-Serine** | **Organic acids and derivatives** | **2.02** | **0.04** | **<0.01** |
| PGF2a ethanolamide | Lipids and lipid-like molecules | 2.01 | 23.63 | <0.01 |

**Table S7**. The relative abundance of bacterial species (top 10 species) at different stages of storage

| **Bacterial species** | **Storage time (days)** | | | | | | |
| --- | --- | --- | --- | --- | --- | --- | --- |
|  | **0** | **2** | **4** | **6** | **8** | **10** | **12** |
| *Bacillus velezensis* | 0.10 ± 0.02^c^ | 0.91 ± 0.84^c^ | 38.96 ± 10.13^ab^ | 30.5 ± 4.98^b^ | 44.15 ± 5.05^a^ | 32.88 ± 12.68^b^ | 35.37 ± 11.36^ab^ |
| *Paenibacillus polymyxa* | 0^a^ | 0^a^ | 0^a^ | 0.01 ± 0.01^a^ | 0.03 ± 0.01^a^ | 0.06 ± 0.08^a^ | 5.32 ± 11.81^a^ |
| *Phaseolus vulgaris* | 15.86 ± 2.36^b^ | 18.67 ± 4.4^a^ | 2.72 ± 1.60^b^ | 2.30 ± 1.34^b^ | 0.51 ± 0.53^b^ | 0.32 ± 0.24^b^ | 0.52 ± 0.37^b^ |
| *Gleditsia sinensis* | 9.52 ± 7.20^a^ | 1.75 ± 1.78^b^ | 1.05 ± 1.14^b^ | 6.08 ± 6.79^ab^ | 2.92 ± 2.08^b^ | 4.89 ± 5.80^ab^ | 4.56 ± 0.48^ab^ |
| *Anoxybacillus sp* | 1.76 ± 0.41^b^ | 3.25 ± 1.59^a^ | 0.25 ± 0.07^c^ | 0.20 ± 0.11^c^ | 0.03 ± 0.03^c^ | 0.01 ± 0.01^c^ | 0.03 ± 0.03^c^ |
| ***Bacillus anthracis*** | **0^b^** | **0.13 ± 0.27^b^** | **0.11 ± 0.16^b^** | **0.32 ± 0.56^b^** | **2.75 ± 2.57^a^** | **0.04 ± 0.06^b^** | **0.02 ± 0.02^b^** |
| *Bacillus aryabhattai* | 0^a^ | 0.02 ± 0.01^a^ | 0.84 ± 1.51^a^ | 0.35 ± 0.32^a^ | 0.19 ± 0.17^a^ | 0.06 ± 0.05^a^ | 0.33 ± 0.30^a^ |
| *Bacillus circulans* | 0^b^ | 0.00 ± 0.01^b^ | 0.73 ± 1.36^ab^ | 0.62 ± 1.01^ab^ | 0.67 ± 0.44^ab^ | 1.65 ± 0.96^a^ | 1.40 ± 0.92^a^ |
| *Psychrobacter alimentarius* | 0.03 ± 0.02^b^ | 0.22 ± 0.41^b^ | 0.01 ± 0.03^b^ | 0.02 ± 0.03^b^ | 0.07 ± 0.14^b^ | 0.07 ± 0.10^b^ | 1.07 ± 1.46^a^ |
| *Acinetobacter johnsonii* | 1.68 ± 0.37^a^ | 1.81 ± 0.49^a^ | 0.16 ± 0.09^b^ | 0.16 ± 0.10^b^ | 0.09 ± 0.03^b^ | 0.08 ± 0.06^b^ | 0.14 ± 0.04^b^ |
| Others | 71.04 ± 7.08^a^ | 73.25 ± 4.52^a^ | 55.17 ± 11.59 ^b^ | 59.44 ± 7.43^b^ | 48.6 ± 6.74^b^ | 59.93 ± 8.98^b^ | 51.24 ± 10.17^b^ |

Values are presented in the table as the mean ± standard deviation.

Means within the different superscript on a row indicate significant differences (*p* < 0.05).
